# Transfer of Maternal Immune Cells by Breastfeeding: Maternal Cytotoxic T Lymphocytes Present in Breast Milk Localize in the Peyer’s Patches of the Nursed Infant

**DOI:** 10.1371/journal.pone.0156762

**Published:** 2016-06-10

**Authors:** Allison Cabinian, Daniel Sinsimer, May Tang, Osvaldo Zumba, Hetali Mehta, Annmarie Toma, Derek Sant’Angelo, Yasmina Laouar, Amale Laouar

**Affiliations:** 1 The Child Health Institute of New Jersey, Robert Wood Johnson Medical School, Rutgers University, New Brunswick, New Jersey, United States of America; 2 Department of Microbiology and Immunology, University of Michigan School of Medicine, Ann Arbor, Michigan, United States of America; INRA, UR1282, FRANCE

## Abstract

Despite our knowledge of the protective role of antibodies passed to infants through breast milk, our understanding of immunity transfer via maternal leukocytes is still limited. To emulate the immunological interface between the mother and her infant while breast-feeding, we used murine pups fostered after birth onto MHC-matched and MHC-mismatched dams. Overall, data revealed that: **1)** Survival of breast milk leukocytes in suckling infants is possible, but not significant after the foster-nursing ceases; **2)** Most breast milk lymphocytes establish themselves in specific areas of the intestine termed Peyer’s patches (PPs); **3)** While most leukocytes in the milk bolus were myeloid cells, the majority of breast milk leukocytes localized to PPs were T lymphocytes, and cytotoxic T cells (CTLs) in particular; **4)** These CTLs exhibit high levels of the gut-homing molecules α4β7 and CCR9, but a reduced expression of the systemic homing marker CD62L; **5)** Under the same activation conditions, transferred CD8 T cells through breast milk have a superior capacity to produce potent cytolytic and inflammatory mediators when compared to those generated by the breastfed infant. It is therefore possible that maternal CTLs found in breast milk are directed to the PPs to compensate for the immature adaptive immune system of the infant in order to protect it against constant oral infectious risks during the postnatal phase.

## Introduction

The mammalian gastrointestinal tract is strongly conserved. For example, the murine and human gut is composed of organs that are anatomically similar. However, both species have some differences. Humans have evolved towards a smaller cecum and colon and relatively longer small intestine as compared to the mouse system [1]. Cells that are essential to intestinal integrity and host-microbiota equilibrium, such as paneth cells, are also conserved between the two species, although there are differences in endogenous components and distribution [2, 3], as well as timing of crypt formation [4]. For instance, in mice, crypt formation starts around day 15 after birth, whereas in humans, mature crypt-villus architecture is already defined at birth [4, 5]. Nevertheless, the gastrointestinal immune system in both species remains immature at birth, since antigenic stimulation of the colonizing microflora is required for its full maturation [1, 4].

Because little antigen exposure occurs in utero, the adaptive immune system of neonates and infants requires considerable education, and this developmental immaturity creates an immunological state of vulnerability for infections in the postnatal period [6–8]. Per definition, T lymphocytes in the intestine of neonates and young infants are considered recent thymic emigrants (RTE), which are progenitors of mature naïve T lymphocytes [9–11]. Compared to adult naïve T lymphocytes, RTEs exhibit functional deficits such as reduced cytokine secretion and cytolytic activity [12, 13]. Mechanisms that facilitate sustained intestinal T cell immaturity during the postnatal period were recently described. Studies found that impaired T cell priming was due to reduced CD28 expression and co-stimulation despite higher TCR and CD3 expressions in these lymphocytes [9]. On the other hand, maternal soluble (S) IgA [14, 15] and neonatal T regulatory _(Reg)_ cells [11, 16] can act in concert to prevent postnatal T lymphocyte maturation under homeostatic conditions. In this regard, breast milk-derived soluble IgA might reduce translocation of luminal antigens previously encountered by the dam, thus preventing immune stimulation of infant T lymphocytes by environmental antigens [14, 15]. Additionally, the secretion of other inhibitory cytokines, such as TGFβ or IL-35, and the indirect inhibitory circuits on DC function via CTLA4 or LAG3 are mechanisms proposed to be involved in T_Reg_-mediated lymphocyte control [11, 16]. However, this active suppression can be a double-edged sword; while it can allow the young intestinal immune system to reinforce self-tolerance by preventing the expansion of lymphocyte clones with ‘neonatal’ reactivity that fail to support host-microbial homeostasis [11], it can be detrimental since the gut is exposed to the environment and the risk of serious infections with exogenous pathogenic microorganisms is constant.

Active immunization through vaccines [13] and passive immunization through breast milk feeding [14–18] are immunological pathways that promote the maturity and development of the infant immune system. Indeed, studies investigating neonatal protection against infection during lactation have shown that breast-feeding provides significant protection to growing offspring against diarrhea caused by *V*. *cholerae*, enterotoxigenic *E*. *coli*, and Campylobacter [17–19]. Additionally, epidemiological data have associated breast-feeding with reduced incidence of immune-mediated diseases such as celiac disease, at least in childhood [20, 21], and diabetes mellitus type I, but these findings need more research [22]. Historically, breast milk has been thought to pass immunity to the growing infant only by virtue of its immunoglobulin content [23–26]. Because breast milk antibodies exist in high amounts and are directed against the microbes the mother has met previously, they are poised to protect the growing infant against a wide array of microorganisms [27, 28]. More recent studies have shown that breast milk also contains additional components of the maternal immune system [29–34]. Indeed, a variety of maternal leukocytes are present in both colostrum and mature breast milk, with macrophages and neutrophils dominating over lymphocytes [34–38]. However, our understanding of the trafficking of these maternal leukocytes and the significance of their transmission to the suckling offspring is still limited. To our knowledge, none of the previous studies [38–45] has investigated the transmission of breast milk leukocytes to offspring’s PPs, the key immunosurveillance regions of the small intestine [46–49]. Additionally, most of the previous studies employed non-physiological protocols of breast milk leukocyte inoculation into starved young infant animals, which exemplify neither the number nor the status of leukocytes physiologically found in breast milk [40, 41, 43, 50].

In the current study, we sought to examine the physiological trafficking of maternal leukocytes to different organs of suckling pups and investigate the transfer of cellular immunity during lactation. Our studies show that maternal leukocytes can be transferred to the pups via breast milk in a number of mouse fostering models. We found that CD8 T cells from the breast milk are taken up by the suckling infants and found in their PPs. A high expression of the gut-homing molecules α4β7 and CCR9 on the milk’s CD8 T cells suggest that this uptake is cell specific. Breast milk is known to suppress rather than increase inflammatory responses in the infant [51–54]. Surprisingly, our investigation showed that transferred breast milk CD8 T cells exhibit functional superiority related to the production of pro-inflammatory and cytolytic mediators when compared to host counterparts. In summary, this study shows that maternal CD8+ T cells found in breast milk home to the PPs of the nursed offspring and may augment cytotoxic T lymphocyte-specific responses of the infant.

## Materials and Methods

### Mice

C57BL/6 (CD45.2), BALB/c, and C57BL/6 (CD45.1) mice were purchased from the Jackson Laboratory in Bar Harbor, Maine. C57BL/6 mice that express a transgene coding for the green fluorescent protein (GFP) of jellyfish under the control of the human ubiquitin C promoter, referred to as GFP^tg^ [55], were also purchased from Jackson Laboratory. Animals were housed in temperature-, water-, and humidity- controlled cages that alternated between 12-h light and dark cycles. All animal experiments were reviewed and approved by the Institutional Animal Care and Use Committee (IACUC) of Rutgers University and Michigan University.

### Fostering of pups

Three experimental models were used, and in all these settings, mice were coordinately mated: 1) C57BL/6 (WT) pups were fostered by C57BL/6 GFP^tg^ dams; 2) C57BL/6 (CD45.1) pups were nursed by C57BL/6 (CD45.2) dams; and 3) BALB/c (H-2b) pups were fostered by C57BL/6 GFP^tg^ (H-2d) dams. Immediately before returning the pups to their cages, we applied a small amount of petrolatum-based decongestant (Vicks^®^ VapoRub^™^) to the snouts of the dams [56]. This step was a cautionary one, in that it aimed to dampen the dam’s sense of smell in order to mask scents the pups may have picked up with handling so that the nursing dams did not reject the fostered pups [56**]**. In general, ~ 80% of fostered pups survived the switch. However, we noticed a decrease in the acceptance of BALB/c pups by C57BL/6-GFP^tg^ dams (~ 50% survival). We do not know the exact explanation of this behavioral phenomenon, but we would assume that the difference in fur color may be a factor. Organs from fostered pups were collected at Weeks 1–3 of nursing and at Weeks 1, 5, and 20 post-weaning for experimental assays.

### Leukocyte Isolation from PPs

Immediately after euthanization, the pup’s intestine was aseptically explanted and placed in a cold complete medium (RPMI 1640 medium containing penicillin (200 IU/mL), streptomycin (200IU/ mL, and 10% fetal bovine serum). Because the yield of leukocytes isolated from PPs is time sensitive, we proceeded with the isolation quickly to obtain better cell viability. PPs were obtained from either a single mouse or combined pups, depending on age. In general, each sample was obtained from a single adult animal (>2 months old); two recently weaned pups (1 month old); three to eight infants (14–20 days old); or eight to ten neonates (< 10 day old). A surgical platform equipped with a clip-on 4x magnifier (Kent Scientific Co, Torrington, CT) was used to harvest infant and neonatal PPs. Collected PPs were then transferred to a 70 μm cell strainer placed over a 60x15 mm Petri dish filled with 5 mL of complete medium supplemented with 100U/mL of collagenase IV (Sigma). The PPs were then dissociated by gently crushing the patches with the plunger of a 1 mL syringe, forcing them through the filter until they were no longer visible pieces of tissue. After 20 min. incubation at 37°C, the filter was washed with 10 mL of complete medium, and the resulting cells were centrifuged at 1100 rpm, at 4°C for 10 min. This mechanical dissociation allowed for the isolation of non-adherent cells, such as B and T lymphocytes, as well as adherent cells, including monocytes and dendritic (both of which require enzymatic treatment to be isolated). To study the phenotype and function of recovered PP T lymphocytes, we limited tissue processing to the mechanical dissociation because enzymatic tissue disintegration can have a profound impact on surface molecule expression and immune function [57]. Moreover, this enzymatic treatment is not required for T cell isolation [58].

### Isolation of hematopoietic cells from the intestinal mucosa and other tissues

PPs were excised; then, the small and large intestine was flushed with PBS and cut longitudinally along its length before being cut into smaller pieces. After three washes, the intestinal pieces were transferred to a 50 mL tube in 20 mL of Hank’s balanced salt solution, which contained 2% FBS and 5 μM EDTA, and then incubated under gentle shaking at 37°C for 20 min. Intraepithelial cells were isolated from the pooled supernatants [58, 59]. Intestinal pieces were then twice digested with 100U/mL of collagenase IV (Sigma) under gentle shaking at 37°C for 20 min. Lamina propria cells were isolated from the pooled supernatants [58, 59]. Intraepithelial and lamina propria cells were pooled together (referred as intestinal mucosa or IM) and stained with antibodies for flow cytometry assays. Other organs such as the spleen (SPL), mesenteric lymph nodes (MLN), and thymus (THY) were processed as followed. Each tissue was transferred to a 70 μm cell strainer placed over a 60x15 mm Petri dish filled with 5 mL of complete medium supplemented with 100U/mL of collagenase IV (Sigma). The tissues were dissociated by gentle crushing with the plunger of a 1 mL syringe. After 20 min. of incubation at 37°C, the filter was washed with 10mL of complete medium, and the resulting cells were centrifuged at 1100 rpm, at 4°C for 10 min. Pellets were resuspended with 3 mL of PBS (MLN and THY) or RBC lysis buffer (SPL) and incubated for 5 min. on ice. Cells were then centrifuged, resuspended in complete RPMI, and reserved for analysis. In some experiments, blood (~ 0.5 mL) was collected from dams through cardiac puncture using 1 mL heparin-coated blood sampling syringes, diluted with 5 mL RBC lysis buffer (Sigma), and incubated for 5 min. on ice. Peripheral blood mononuclear cells were then centrifuged, washed twice with the complete medium, and analyzed for flow cytometry.

### Isolation of leukocytes from the milk bolus

Milk bolus was extracted from the stomach of a one-week old pup (~3 to 5 milk boluses were combined per sample), transferred in a 100 μm cell strainer placed over a 60x15 mm Petri dish filled with 5 mL of complete medium supplemented with 100U/mL of collagenase IV (Sigma). The milk bolus was dissociated by crushing milk curd particles with the plunger of a 1 mL syringe through the filter until they were no longer visible milk curds. After 20 min. of incubation at 37°C, the filter was washed with 45 mL of 0·17 M Tris (pH 7·6) and 0·83% NH_4_Cl buffer [60] and centrifuged at 400 × *g* at 20°C for 15 min. Pellets containing maternal leukocytes were transferred to a 2 mL eppendorf tube and washed four times with the complete medium to remove the majority of the fat and the whey prior to further analysis.

### Flow Cytometry

Cells were first stained with 2.4G2 (eBioscience) to block Fc receptors. They were then surface labeled with antibodies against mouse CD45.1, CD45.2, CD3, CD4, CD8, CD11b, CD11c, CD19, Gr-1, α4β7, CCR9, and/or CD62L (BD Biosciences). Subsequently, each sample was stained with 5 μL of fluorochrome-conjugated Annexin V and 5 μL of Propidium Iodide Staining Solution (Affymetrix Bioscience), and then analyzed by flow cytometry within 4 hours in the dark. In some experiments, cells were stained intra-cellularly with anti-mouse TNFα, IFNγ, IL-18, and Granzyme B (eBioscience), using the Cytofix/Cytoperm Kit (BD Biosciences). Flow cytometry was performed on an LSR II (BD Biosciences) using FACSDiva software (BD Biosciences). Before conducting the sample analysis, the flow cytometer settings were checked using Cytometer Setup and Tracking beads (CS&T beads, BD) according to the manufacturer’s instructions. Compensation beads were used with single stain of each antibody to determine the compensation settings, and these were applied in the FlowJo software (version 10.0.6, Tree Star, Ashland, OR, USA) after data collection. In addition, doublets were excluded by using a forward scatter (FSC)-H vs. FSC-A plot. A side scatter (SSC) threshold level was set at 5,000 units to eliminate debris, and at least 5,000 cells were counted. The same compensation matrix was applied to all samples. Recorded data were compensated post-hoc and analyzed using FACSDIVA, CellQuest (BD Biosciences), and FlowJo (Tree Star) software.

#### Gating Strategy

**Fig 1:**
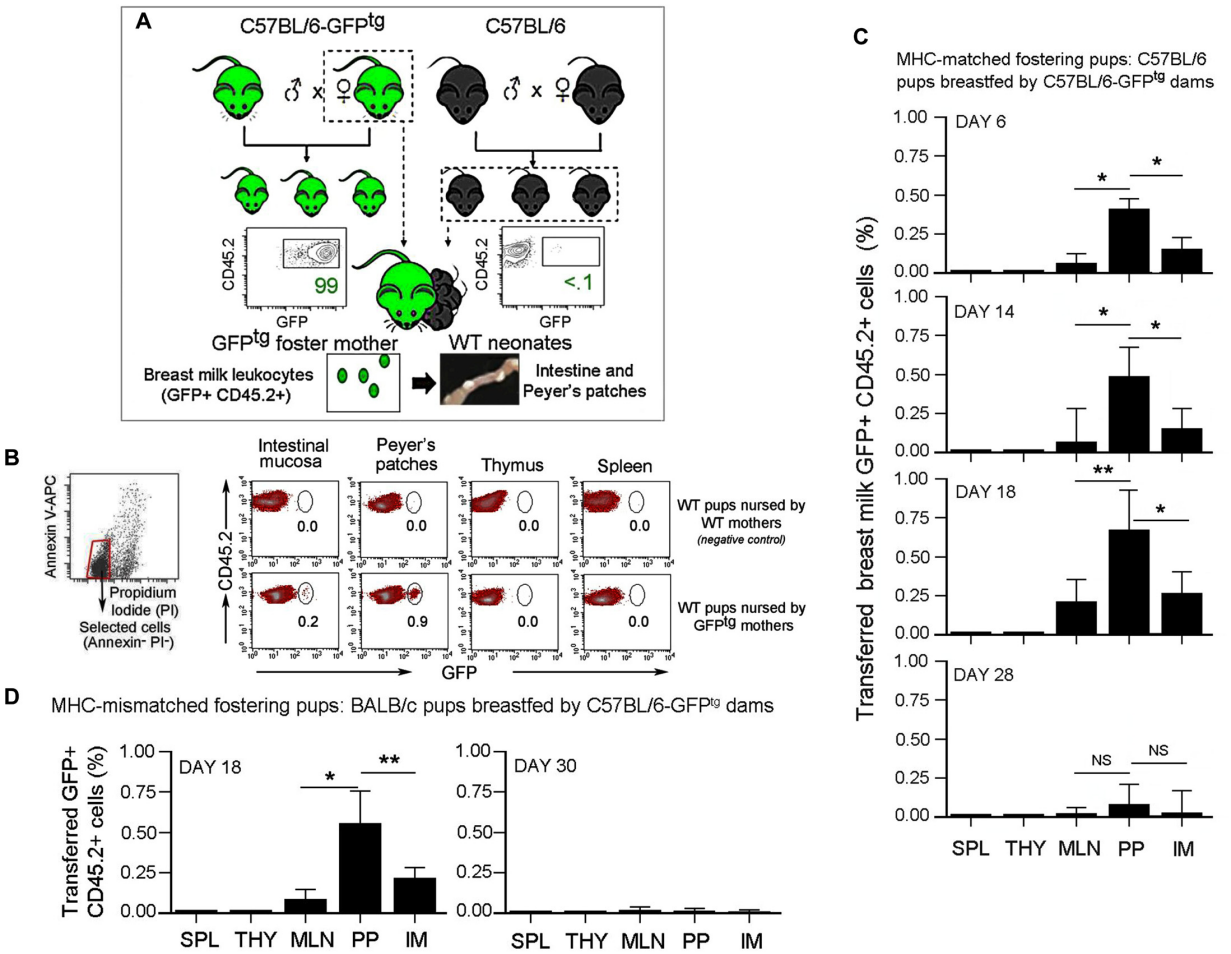
Transfer of breast milk leukocytes to suckling pups. **(A)** Breeding of wild type (WT) C57BL/6 and C57BL/6-GFP^tg^ (GFP^tg^) mice was coordinately mated. At day 0–2, WT neonates were transferred to be continually nursed by GFP^tg^ dams until their weaning (21 days). PPs and other organs from suckling infants were surgically excised, purified, and put into cell suspensions for flow cytometry analysis. FACS plots show the level of GFP expression by CD45.2+ splenocytes of GFP^tg^ (*left*, positive control) and WT (*right*, negative control) mice. **(B)** Leukocytes were identified using the following orientating gates: The first orientating gate was selected using a SSC vs. CD45 plot where the cutoff for CD45+ cells was set using FMO control. Subsequently, annexin V vs. propidium iodide (PI) plot of CD45+ cells separated events into a major gate of annexin V^-^ PI^-^ events which represents viable CD45+ cells. Subsequent specific gate for other populations that were preselected as CD45+ annexin V^-^ PI^-^ are shown in CD45 vs. GFP plots. Dot Plots show the presence of breast milk leukocytes (GFP+CD45.2+) in the peyer’s patches (PPs), spleen (SPL), thymus (THY), mesenteric lymphnodes (MLN), and intestinal mucosa (IM). Percent of transferred leukocytes was assessed 18 days post-fostering. Panels show WT pups breastfed by WT mothers (*upper*, *negative control*) and WT pups breastfed by MHC-matched GFP^tg^ dams (*lower*). **(C)** Bar graphs summarize the presence of GFP+ cells in organs as a % of total CD45.2+ cells at day 6, 14, and 18 post-fostering, and one week post-weaning (day 28). Data were obtained from three experiments using a total of 24 pups with *n* = 6–10 pups per experiment (DAY 6); three experiments using a total of 16 pups with *n =* 4–6 pups per experiment (DAY 14); five experiments using a total of 26 pups with *n =* 4–6 pups per experiment (DAY 18); and three experiments using a total of 9 mice with *n = 3* animals per experiment (DAY 28). **(D)** BALB/c neonates were transferred to be foster-nursed by GFP^tg^ dams until their weaning. Bar graph summarizes the presence of GFP+ cells as a % of total CD45.2+ cells in organs at day 18 post-fostering and one week post-weaning (day 30). Data were generated from three experiments using a total of 12 pups (DAY 18) and 6 mice (Day 30) with *n* = 2–5 animals per experiment. **(C and D)** Data are shown as means of % GFP+ CD45.2+ cells ± s.e.m. Error bars represent s.e.m. A 2-tailed Student’s *t* test distribution with paired groups was evaluated for statistical significance. P > 0.05 was considered not significant (NS), *P < 0.05 was considered significant, and **P < 0.005.

**Fig 2:**
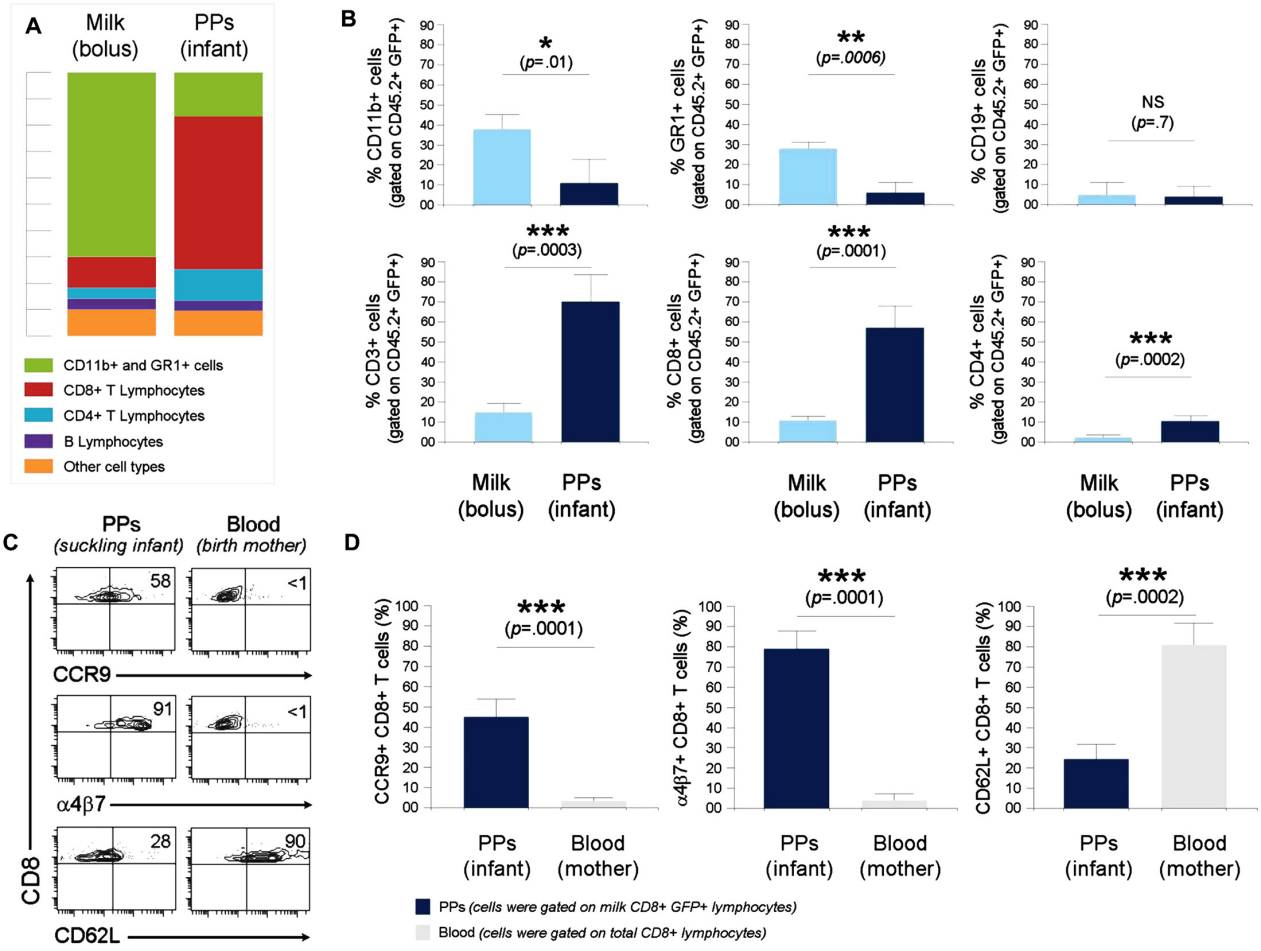
Characterization of breast milk leukocytes. **(A)** Different GFP+ cell subtypes were identified in milk bolus and PPs of C57BL/6 pups nursed by GFP^tg^ dams based on the co-expression of GFP and CD45.2 in combination with CD3, CD8, CD4, CD11b, CD19, and Gr1. Bar graphs show the average contribution of cell subset(s) to the total of GFP+CD45.2+ cells in milk bolus (*left*) and PPs (*right*). **(B)** Bar graphs show the % of each cell subset (gated on total CD45.2+ GFP+ cells) in milk bolus versus PPs: CD11b+ (CD3-CD19-CD11b+, *upper left*), GR1+ (CD3-CD19-Gr-1+, *upper middle*), CD19+ (CD3-CD19+, *upper right*), CD3+ (CD19-CD11b-CD3+, *lower left*), CD8+ (CD3+CD8+, *lower middle*), and CD4+ (CD3+CD4+, *lower right*) cells. Data are shown as means of % cell subset gated on GFP+CD45.2+ cells ± SD. Error bars represent SD. **(A and B)** Data were obtained from four experiments using a total of 16 (milk bolus) and 25 (PPs) animals with a combined material of 3 to 5 (milk) and 5 to 8 (PPs) mice per experiment. (**C**) Contour Plots show expression of α4β7, CCR9, and CD62L by breast milk CD8+GFP+ T cells that localize in PPs of suckling pups vs. blood CTLs of the birth mother. (**D**) Bar graphs show the % of CD8+CCR9+, CD8+α4β7+, and CD8+CD62L+ in PPs (gated on milk CD3+CD8+GFP+ cells) versus adult blood (gated on total CD3+CD8+ cells). Data are shown as means of % cell subset gated on CD8+GFP+ (PPs) or CD8+ (blood) T lymphocytes ± SD. Error bars represent SD. Bar graphs summarize the data from four experiments using a total of 25 (PPs) and 5 (blood) animals with a combined material of 5 to 8 (PPs) and 1 to 2 (blood) mice per experiment. A 2-tailed Student’s *t* test distribution with paired groups was evaluated for statistical significance. *P < 0.05 was considered significant, **P < 0.005, ***P < 0.0005, and P > 0.05 was considered not significant (NS).

**Fig 3:**
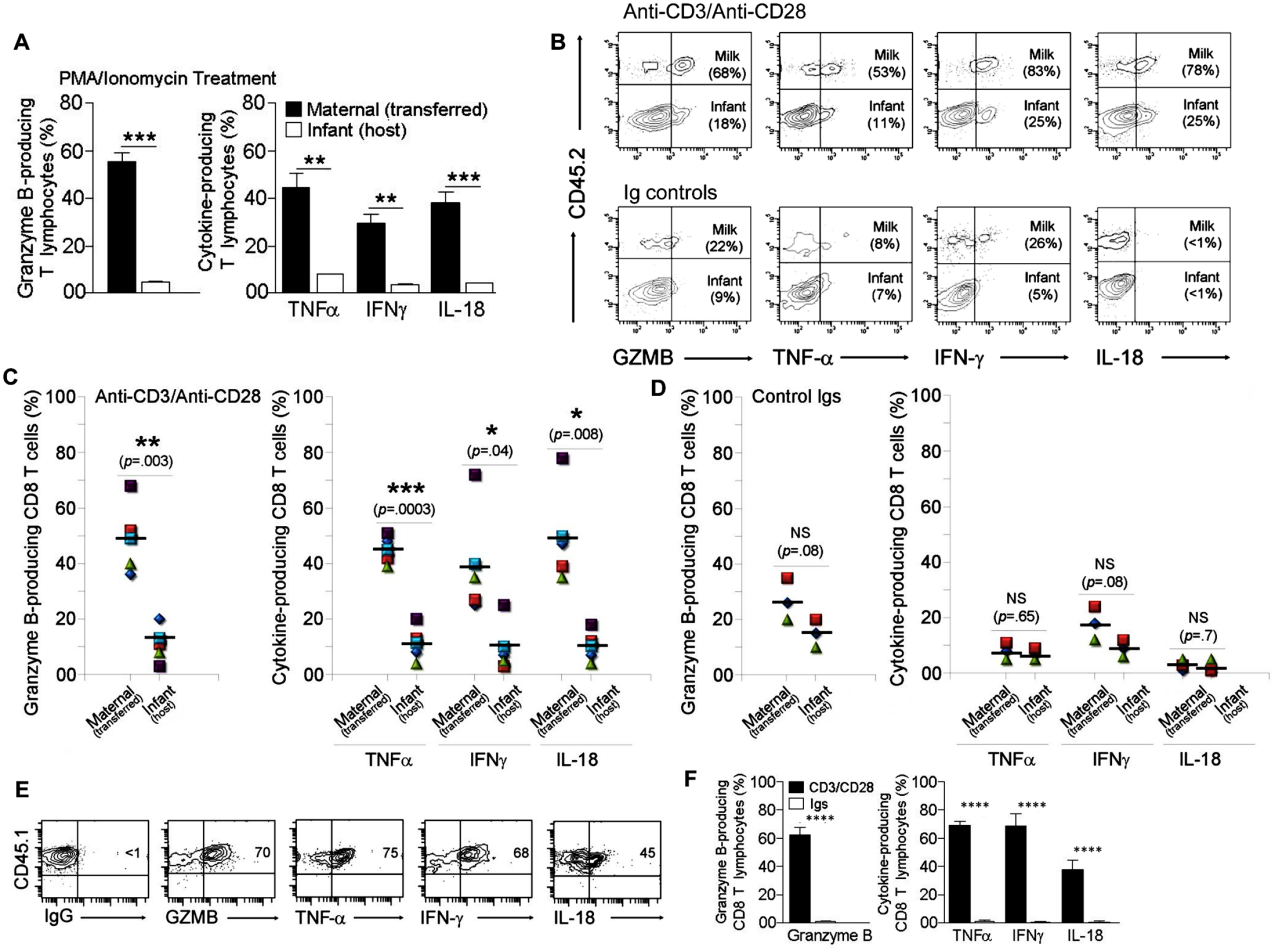
Production of granzyme B and pro-inflammatory cytokines by breast milk and infant T cells. PPs from CD45.1 pups breastfed by CD45.2 congenic dams for 18 days were collected, put into cell suspensions, and processed for T cell purification. **(A)** Enriched T lymphocytes were stimulated overnight with PMA and ionomycin. Bar graphs show % cytokine- and GRMB-producing maternal cells (transferred milk CD45.2+) vs. infant (host CD45.2-) T lymphocytes. Data were obtained from three experiments shown as means of % CD3+ T cells ± s.e.m. Error bars represent s.e.m. **P < 0.005, ***P < 0.0005 using student’s two-tailed t test. **(B-D)** Enriched T lymphocytes were activated with anti-CD3 /anti-CD28 Abs or control Igs for four days in the presence of 200U/mL of IL-2. T cell responses associated with IFN‐γ, TNF-α, Granzyme B (GZMB), and IL-18 production were examined by intracellular staining and analyzed by FACS. The first orientating gate was selected using a SSC vs. CD3 plot where the cutoff for CD3+ cells was set using FMO control. Subsequent gating of a CD3 vs. CD8 plot of total CD3+ cells separated events into a selected population of CD3+CD8+ cells. Subsequent specific gates identified infant (host CD45.2-) and maternal (transferred breast milk CD45.2+) cells that were producing cytokines and GZMB. **(B)** Contour Plots show outcomes from activation assays with anti-CD3/anti-CD28 (*upper panel*) and isotype (*lower panel*) Abs. Graphs show functional outcomes **(C)** with anti-CD3/anti-CD28 Abs (*n* = 5) and **(D)** isotype controls (*n* = 3). The expression of each product was shown as means of % CD45.2+ CD8+ (maternal) or CD45.2- CD8+ (infant) T cells ± s.e.m. (**B-D)** A 2-tailed Student’s *t* test distribution with paired groups using equal number of samples was evaluated for statistical significance. *P < 0.05 was considered significant, **P < 0.005, ***P < 0.0005, and P > 0.05 was considered not significant (NS). **(E)** Peripheral blood CD8 T cells collected from birth mothers (CD45.1+) were activated with anti-CD3/anti-CD28 Abs. Four days later, cytokine and GZMB production was measured as in b-d. FACS Plots show CD8+CD45.1+ cells that were producing IFN‐γ, TNF-α, GZMB, and IL-18. **(F)** Bar graph summarizes the data from three experiments using two dams per experiment. Data are shown as means of % CD8+ CD45.1+ cells ± SD. Error bars represent SD. ****P < .0005 using a 2-tailed Student’s *t* test.

The leukocytes of the milk bolus and organs were identified based on the methods of Faucher et al. by using similar orientating and specific gates as previously described [61]. The first orientating gate was selected using a SSC vs. CD45 plot, where the cutoff for CD45+ cells was set using an FMO control. Subsequently, an annexin V vs. propidium iodide (PI) plot of CD45+ cells separated events into a major gate of double negative annexin V^-^ PI^-^ events, which represents viable CD45 cells, and other orientating PI^+^ and/or annexin V+ dead cell populations. Subsequent orientating and specific gates for other populations (preselected as CD45+ annexin V^-^ PI^-^) are shown in Figs 1–3. This gating strategy was used to identify viable leukocyte types such as myeloid cells (CD11b), B lymphocytes (CD19), cytotoxic T cells (CD8), helper T cells (CD4), and granulocytes (Gr1) in the milk bolus and PPs of foster-nursed infants. The overall assessment of cell viability by PI and annexin V staining showed variability in the percentage of viable leukocytes recovered from MHC-matched (75±11) and MHC-mismatched (51±19) fostered pups with a *p-value of* 0.09. This variability could be due to MHC-mismatched fostering and/or other experimental factors, such the time elapsed between the euthanization and the processing of the cells for FACS.

### Activation of infant and breast milk T lymphocytes

Using synchronized breeding of CD45.1 and CD45.2 mice, the age-matched pups were fed with switched mothers for 18 days. In this experimental model, the use of the CD45.2 strain enabled the maternal T cells to be traced in the CD45.1 offspring. PPs from CD45.1 pups fed by CD45.2 dams were collected, made into cell suspensions, as described above, and processed for T cell purification using nylon wool separation columns. This is an efficient method for intestinal T cell isolation as it requires no further cell processing and thus eliminating the risk of altering the phenotype and/or the function of selected T lymphocytes [58, 59, 62]. Purified cells were diluted at 1–10 x10^5^ cells/mL in the complete medium supplemented with 2 X of Pen/Strep to avoid a possible bacterial contamination. The cells were then placed in a 37°C and 5% CO_2_ incubator overnight with PMA (5 ng/mL; Sigma) and ionomycin (500 ng/mL; Sigma) in the presence of the protein transport inhibitor GolgiStop (BD Bioscience). In parallel experiments, T cells were activated with anti-CD3/anti-CD28 mAbs or isotype Igs at 1 μg/mL and 5 μg/mL final concentrations, respectively. Additionally, we tested blood CD8 T cells of birth mothers to determine whether transferred milk CD8 T cells are functionally different from average circulating adult CTLs when responding to TCR/CD3 stimulation. On the third day, cultures were supplemented overnight with GolgiStop for intracellular staining. All samples were washed in cold PBS supplemented with 2% FBS and stained with 2.4G2 (eBioscience) to block Fc receptors. They were then surface labeled with antibodies to mouse CD45.1, CD45.2, CD3, and/or CD8. Subsequently, cells were stained intracellularly with anti-mouse TNFα, IFNγ, IL-18, or Granzyme B using the manufacturer’s (BD Biosciences) suggested Cytofix/Cytoperm Kit. The cells were then analyzed by flow cytometry. The first orientating gate was selected using a SSC vs. CD3 plot, where the cutoff for CD3+ cells was set using an FMO control. For some samples, the subsequent gating of a CD3 vs. CD8 plot with a total of CD3+ cells separated events into a selected population of CD3+CD8+ cells. Subsequent specific gates for other populations are shown in Fig 3.

### Statistics

Microsoft Excel for PC and Prism for Mac (GraphPad, La Jolla CA, USA) software programs were used to calculate the arithmetic mean, standard error of the mean, and/ or standard deviation for each group of animals. The 2-tailed Student’s *t* test distribution with paired (*p1*) and/or two-sample equal variance (*p2*) was used. *P* < 0.05 was considered significant.

## Results

### Breast milk leukocytes localize in the Peyer’s patches of suckling pups

To track the passage of breast milk leukocytes to suckling infants, we utilized GFP^tg^ dams that continually foster-nursed wild type (WT) pups immediately postpartum (Fig 1A and S1 Fig). As anticipated, GFP+ cells were absent in WT pups that were nursed by WT mothers (Fig 1b), but they were found in the immune systems of GFP pups grown in GFP^tg^ mothers during normal pregnancy (Fig 1a) as well as WT pups that had been fed by GFP^tg^ foster dams (Fig 1b). Our data were recorded using a side scatter (SSC) threshold level of 5,000 units to eliminate debris. Importantly, the gating strategy was set to exclude all apoptotic cells by positively selecting only viable CD45+ cells that were negative for PI and/or annexin V (Fig 1b). Importantly, our data revealed the preferential presence of breast milk GFP+ leukocytes in the PPs of suckling pups (Fig 1B and 1C). This phenotype was evident at 14–18 days of age when the detection of milk GFP+ cells reached its maximum (Fig 1B and 1C). Interestingly, only very few GFP+ cells were detected in other tissues (Fig 1B and 1C), suggesting that most transferred milk leukocytes localize in the PP of suckling pups. However, once breastfeeding by GFP+ dams ceased, the GFP+ cells were detected neither in newly weaned (1 week post-weaning) nor in adult (5 and 20 weeks post-weaning) animals (Fig 1C and data not shown).

In humans, the MHC in mothers is not compatible with that of their offspring because of the duality of genetic chromosomal determination from mother and father. Therefore, we extended our investigation to examine the transfer of breast milk leukocytes into the circulation of MHC-mismatched pups. Briefly, BALB/c (H2-d) neonates were foster-nursed immediately postpartum by C57BL/6-GFP^tg^ (H2-b) mothers (S2 Fig). Using similar strategies described in Fig 1a and S1 Fig, the GFP+ cells were analyzed in BALB/c pups at eighteen days following the commencement of foster nursing. In agreement with our previous findings, the major presence of GFP+ cells also occurred in the PPs of breastfed pups (Fig 1d). Additionally, tissues from weaned BALB/c animals (30 days of age) previously foster-nursed by GFP^tg^ dams were examined to determine the longevity of breast milk GFP+ cells. In BALB/c mice examined for this purpose, PPs and other tissues contained little or no GFP+ cells (Fig 1d). Therefore, long-term survival of milk leukocytes in MHC-mismatched infants is possible, but not significant after the breast feeding ceases. Collectively, these results show the transmission of maternal leukocytes to MHC-matched and MHC-mismatched offspring by breast milk feeding, and reveal the preferential localization of transferred maternal leukocytes in the PPs of suckling pups.

### Characterization of breast milk leukocytes that localize in the PPs of suckling infants

Previous data suggest that maternal leukocytes can be transferred to the PPs of suckling pups by breast milk feeding (Fig 1). However, the leukocyte types being transferred were unknown. Prior to investigating this issue, we first characterized the composition of leukocyte types in breast milk. To this end, milk boluses extracted from the stomachs of nursed pups by GFP^tg^ dams were co-stained with antibodies that recognize different immune cell types. For data acquisition and analysis, we used orientating and specific gates as described in Methods and S3A Fig. Our data revealed that most GFP+ leukocytes in milk bolus were myeloid cells (Gr-1+, CD11b+, and CD11c+, 80±15%), while lymphocytes (CD8+, CD4+, and CD19+) constituted only 20±10% of total CD45+GFP+ cells (Fig 2A, 2B and S3A Fig). Remarkably, most GFP+ leukocytes localized in the PPs were neither myeloid nor B cells, as determined by CD11b, CD11c, Gr-1, and CD19 co-staining (Fig 2a and 2B). Further investigation showed that the majority of transferred GFP+ cells to be T lymphocytes, and predominantly CD8 T cell type (Fig 2A and 2B, S3B and S3C Fig). Thus, while most leukocytes in the milk bolus were myeloid cells, the majority of breast milk cells localized in infant PPs were cytotoxic T cells (CTLs). We hypothesized that this outcome could be attributed to the expression of gut-homing molecules directing milk CD8 T cells to host PPs. Indeed, our data revealed that contrary to circulating adult CD8 T cells, most breast milk CTLs that sequester in infant PPs exhibit high levels of the gut-homing integrin α4β7 and chemokine receptor CCR9, but have a reduced expression of the systemic homing marker CD62L (Fig 2C and 2D). These findings may explain why breast milk CD8 T cells were preferentially directed to the PPs, and suggest that this uptake is cell specific. Of note, we were not able to assess these markers on other transferred milk cell types due to their limited numbers in neonatal and infant PPs. Overall data suggest that breast milk CD8+ T lymphocyte uptake by infant PPs is cell specific.

### Outcomes of the concomitant activation of infant and breast milk cytotoxic T lymphocytes

After observing the transfer of breast milk CD8 T cells to suckling pups, we investigated the functional significance of this transfer on T cell-mediated inflammatory functions. T cells can be stimulated in various ways to produce effector mediators [63, 64]. In this study we used two common methods for the induction of cytokines by T lymphocytes: the mitogenic stimulation with phorbol 12-myristate 13-acetate (PMA) and ionomycin [64, 65] and a more physiological approach using anti-CD3 and anti-CD28 Abs that mimic stimulation by antigen presenting cells [65, 66]. Generally, congenic mice with CD45.1 vs. CD45.2 alleles are used in transfer models to discriminate between donor and recipient T lymphocytes [67, 68]. Therefore, we employed these two lines and conducted activation assays to test whether production levels of cytolytic and inflammatory mediators would be altered in transferred breast milk T cells when compared to those generated by the offspring counterparts. To ensure that we are being accurate in our comparison as well as to avoid the scenario that breast milk T cells respond to pup self-antigen, we used MHC-matched animals. Briefly, using synchronized breeding of the MHC-matched CD45.1 and CD45.2 mice, the age-matched pups were fed with switched mothers for 18 days. Subsequently, the PPs of foster-nursed pups were collected and processed to isolate T cells, which were then stimulated with PMA/Ionomycin or activated with anti-CD3/anti-CD28 mAbs. Because T cell responses associated with IFN‐γ, TNF-α, granzyme B (GZMB), and IL-18 production play central roles in host defense against harmful pathogens [69–71], we examined the efficiency of infant vs. transferred breast milk T cells at producing these inflammatory molecules. Our data showed that the production of inflammatory cytokines and GZMB elicited by activation with PMA/Ionomycin (Fig 3A) and anti-CD3/anti-CD28 Abs (S3D Fig) of breast milk T lymphocytes were significantly increased when compared to those of infant T cells. To ensure that we are being accurate in our comparison between infant and breast milk cells, we extended our study to analyze exclusively CTLs. Control experiments with isotype Abs showed little or no production of inflammatory mediators by infant and transferred maternal CTLs (Fig 3B and 3D). Importantly, the levels of inflammatory cytokines and granzyme B elicited by activated milk CTLs were on average four times higher when compared to those generated by the infant counterparts (Fig 3B and 3C), but they were closely similar to those produced by the circulating adult CD8 T cells of the birth mother (Fig 3E and 3F). Together, these findings suggest that in comparison to infant CD8 T cells, transferred breast milk counterparts are more potent at producing cytolytic and inflammatory mediators and may augment CTL-specific responses of the infant.

## Discussion

The interaction between mother and infant via breast milk postpartum plays an important role in the development of the infant immune system [6, 14, 72, 73]. Breast milk contains antibodies, cytokines, and other proteins that help to fight microorganisms once ingested by the infant [74, 75]. Indeed, when compared with formula-fed infants, breast milk has been associated with decreased incidence of infectious diseases such as gastroenteritis [76] and urinary tract infections [77]. A great focus has been dedicated to the importance of breast milk antibodies passed to suckling neonates [14, 78]. However, as is true of defensive antibodies and other molecules, immune cells are also present in breast milk [40, 41, 43, 50, 79].

Pioneering studies investigating the transfer of immune cells via breast milk have been performed by other groups [38–45]. However, these groups have not studied maternal cell transfer to offspring PPs. These are crucial anatomical regions of the intestine that are involved in immune surveillance of pathogenic microbes entering the intestinal tract [46–48]. Additionally, most of the previous studies employed radiolabeled and/or concentrated breast milk leukocytes that were orally inoculated into starved animals [40, 41, 43, 50]. However, these methods do not study the cellular transfer physiologically, because oral inoculation of manipulated and/or concentrated leukocytes represents neither the concentration nor the status of leukocytes normally found in breast milk. In the current study, we sought to examine the transfer of maternal cells in a physiological manner. Two other groups have examined physiological transfer of breast milk leukocytes using similar approaches [60, 80]. However, no phenotypical and immunological findings of cell transfer to the PPs of suckling pups were investigated. Moreover, these studies did not examine the physiological transfer in MHC-mismatched fostering settings that mimic the human breast-feeding events, since human mothers do not share the same MHC compatibility as their offspring. In our studies, MHC-matched as well as MHC-mismatched pups were fostered 0–2 days after birth onto GFP^tg^ dams and remained there until Week 3 when separated for weaning. This approach allowed us to track transferred maternal cells in different tissues and examine their phenotypes in practicable detail. Our study revealed that hematopoietic-derived cells from the breast milk survive the infant gastrointestinal tract and remain until weaning. These observations suggest that a reasonably hospitable environment, probably due to weak digestive enzyme activities and low acidity of the infant stomach [79, 81–83], persists during the course of breast-feeding, allowing for the survival of maternal milk leukocytes. Surprisingly, most maternal leukocytes establish themselves in specific areas of the intestine, the PPs. One explanation for this phenotype could be that, when compared to the rest of the intestinal mucosa, the epithelial surface of PPs is more permeable and accessible to luminal content, which may facilitate maternal cell transfer during suckling [46, 47, 84]. Importantly, this phenotype was reproducible in MHC-mismatched animals (a set-up that emulates the immunological interface between the human mother and her infant while breast-feeding), suggesting that breast milk leukocytes may target the infant PP independent of the MHC haplotype. This observation has yet to be fully explained. However, it is possible that the offspring becomes tolerant to the maternal major histocompatibility antigens due to its immunological developmental immaturity and/or the massive presence of TGFβ in breast milk that may actively prevent the expansion of lymphocyte clones with maternal reactivity [11, 38, 79, 85, 86].

Breast milk contains large amounts of myeloid cells, such as macrophages and granulocytes [35, 36]. In this context, our data also revealed that most leukocytes in murine milk bolus were myeloid in nature. Interestingly, our studies did not show significant transfer of milk myeloid cells. There is some evidence in the literature for the transfer of these cells, but the physiological relevance of these observations remains unclear [87, 88]. Our data instead showed that most transferred cells to the intestine during physiological suckling were T cells, in spite of much higher numbers of other cell types in the milk bolus. In our studies, T cells made up about 80% of the cells being transferred, and the proportions of each cell type within the transferred T cell population was determined as ~25% CD4+ and ~75% CD8+. The origin of breast milk CD8 T lymphocytes is uncertain. The mammary glands are a uniquely designed extension of the mucosal immune system of the gut, and therefore lactation links the suckling infant irrevocably to the immunologic and infectious experience of its nursing mother [89]. There are few data on T lymphocyte migration between the maternal intestine and the lactating breast. Labeling studies in female rats and pigs showed that lymphocytes from PPs migrate specifically to the lactating mammary gland [90–95] and that pregnancy increases the mucosal vascular addressing MadCAM-1 expression in the breast, which interacts with the gut-homing receptor α4β7 [96]. In line with these observations, we find that most transferred milk CD8 T cells to the PPs exhibit high levels of the gut integrin α4β7 and chemokine receptor CCR9, but a reduced expression of the systemic homing marker CD62L [97]. Such a phenotype strengthens the argument that this cell uptake is specific and may explain why breast milk CTLs were preferentially directed to the PPs.

What could be the role of the transferred breast milk CTLs? The neonatal innate immune system is present from birth and capable of protection at the local level, but the adaptive immune system is initially naïve and needs considerable time to mature and generate appropriate inflammatory responses [79, 85, 86, 98]. Therefore, it is possible that maternal CD8 T cells are directed to the PPs to compensate for this relative immune immaturity by increasing inflammatory responses of the infant. Indeed, our investigation showed that in comparison to infant CD8 T cells, transferred breast milk counterparts exhibit functional superiority related to the production of inflammatory and cytolytic molecules. We think that these findings may have a significant relevance to the immunological needs and protection of infants during the postnatal phase, but this requires more work to be done on the mechanisms through which transferred milk CTL to PPs may influence the outcome of infectious diseases in the suckling offspring. As an example, breast-feeding infants of human immunodeficiency virus (HIV)-infected women ingest large amounts of HIV, but most escape infection because breast milk cellular responses are potentially influential in decreasing mother-to-child transmission of viruses [99]. In particular, the presence of HIV-specific major histocompatibility complex class I-restricted CD8+ CTLs in breast milk suggests a role in limiting transmission [32]. In this context, investigating the function of transferred breast milk HIV-specific CTLs to neonatal and infant PPs may provide a rationale for mucosal vaccine strategies to enhance these responses.

To conclude, this study confirms maternal leukocyte transmission to offspring by breast milk feeding after birth. While most maternal leukocytes in the milk bolus are of myeloid lineage, transferred cytotoxic T lymphocytes preferentially migrate to the PPs of breastfed infant. Their site-specific distribution, probably facilitated by high expression of the gut-homing molecules α4β7 and CCR9, as well as immunological competence are likely to contribute to the support of the deficient mucosal CTLs of growing infants. In this regard, future studies utilizing confocal microscopy as well as *in vivo* infection models using TCR transgenic and Rag-deficient mice will further characterize these breast milk CTLs and define their role in protecting against pathogenicity of oral infections, and their impact on the development of pediatric diseases.

## Supporting Information

S1 Fig Schematic presentation of the experimental use of MHC-matched GFP^tg^ dams and WT pups. Breeding of wild type C57BL/6 (WT) and C57BL/6-GFP^tg^ (GFP^tg^) mice was coordinately mated. At day 0–2, WT neonates were transferred to be continually nursed by GFP^tg^ or WT (control) dams until their weaning. Kinetic data were obtained from three to five experiments with *n* = 9 to 26 animals per time point.
(TIF)

S2 Fig Schematic presentation of the experimental use of MHC-mismatched C57BL/6-GFP^tg^ (H2-b) dams and BALB/c (H2-d) pups. BALB/c neonates were transferred to be continually breastfed by GFP^tg^ or BALB/c (control) dams until their weaning. Data were obtained from three experiments using a total of 13 (DAY 18) and 6 (DAY 30) animals.
(TIF)

S3 Fig Characterization of breast milk leukocytes. (**A**) Using similar orientating and specific gates as previously described by Faucher et al., [61], leukocyte types such as myeloid cells (CD11b) and T lymphocyte (CD3) subsets were identified in milk bolus. **(B)** Bar graphs show the average contribution of each T cell subset to CD3+GFP+T cells in milk bolus and PPs. **(C)** Bar graphs show the % of each T cell subset gated on total CD3+ GFP+ cells in milk bolus vs. PPs. Data are shown as means of % cell subset gated on CD3+GFP+ T cells SD. Error bars represent SD. Data were obtained from 4 experiments using a total of 16 (milk bolus) and 25 (PPs) animals with a combined material of 3–5 (milk) and 5–8 (PPs) mice per experiment. (**D**) PPs from CD45.1 pups foster-nursed by CD45.2 congenic dams for 18 days were collected, put into cell suspensions, and processed for T cell purification. Enriched T lymphocytes were activated with anti-CD3/anti-CD28 Abs for four days in the presence of 200U/ml of IL-2. T cell responses were examined by intracellular staining and analyzed by FACS. Bar graphs show % granzyme B- and cytokine-producing maternal (transferred by breast milk feeding, black) vs. infant (host, white) T lymphocytes. Data obtained from three experiments are shown as means of CD3+ T cells ± s.e.m. Error bars represent s.e.m. *P* < 0.05 was considered significant, **P < 0.005, ***P < 0.0005using student’s two-tailed t test.
(TIF)
