# Supplementary figures and images for: Transfer of Maternal Immune Cells by Breastfeeding: Maternal Cytotoxic T Lymphocytes Present in Breast Milk Localize in the Peyer’s Patches of the Nursed Infant
Source: PLoS One. 2016 Jun 10;11(6):e0156762. doi: 10.1371/journal.pone.0156762 (PMC4902239; doi:10.1371/journal.pone.0156762)

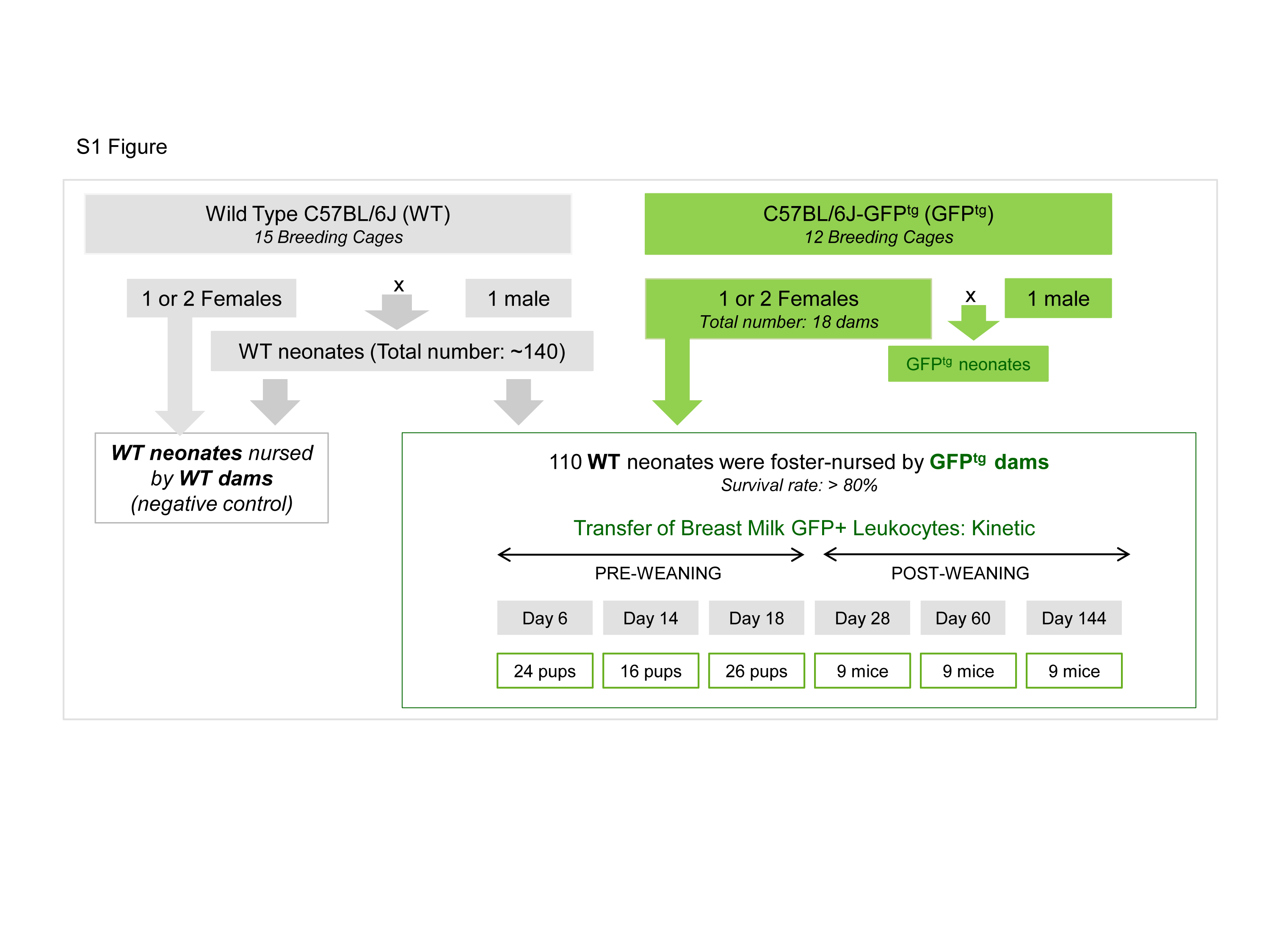

Supplement: S1 Fig — Breeding of wild type C57BL/6 (WT) and C57BL/6-GFPtg (GFPtg) mice was coordinately mated. At day 0–2, WT neonates were transferred to be continually nursed by GFPtg or WT (control) dams until their weaning. Kinetic data were obtained from three to five experiments with n = 9 to 26 animals per time point. (TIF) [file pone.0156762.s001.tif]

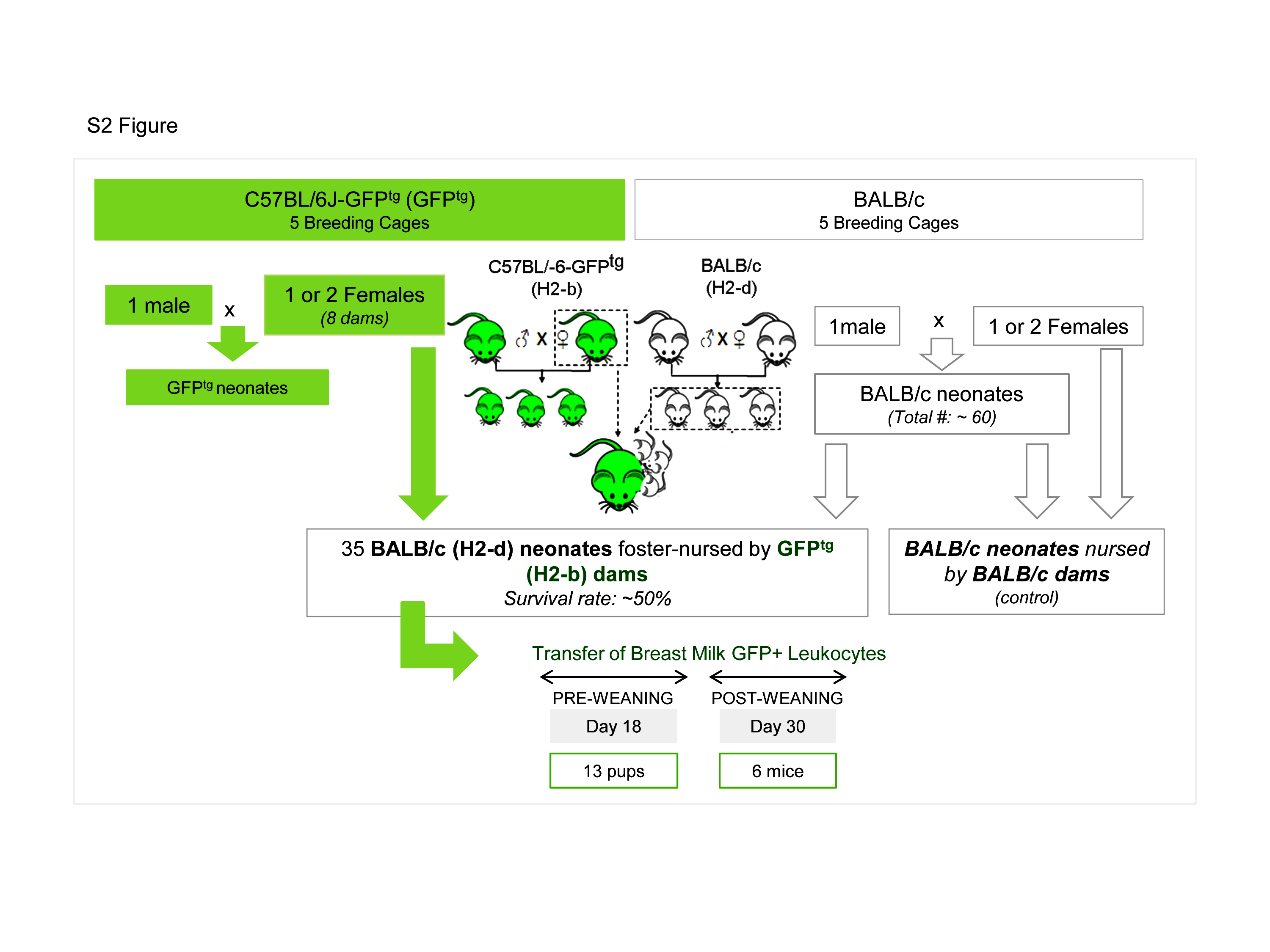

Supplement: S2 Fig — BALB/c neonates were transferred to be continually breastfed by GFPtg or BALB/c (control) dams until their weaning. Data were obtained from three experiments using a total of 13 (DAY 18) and 6 (DAY 30) animals. (TIF) [file pone.0156762.s002.tif]

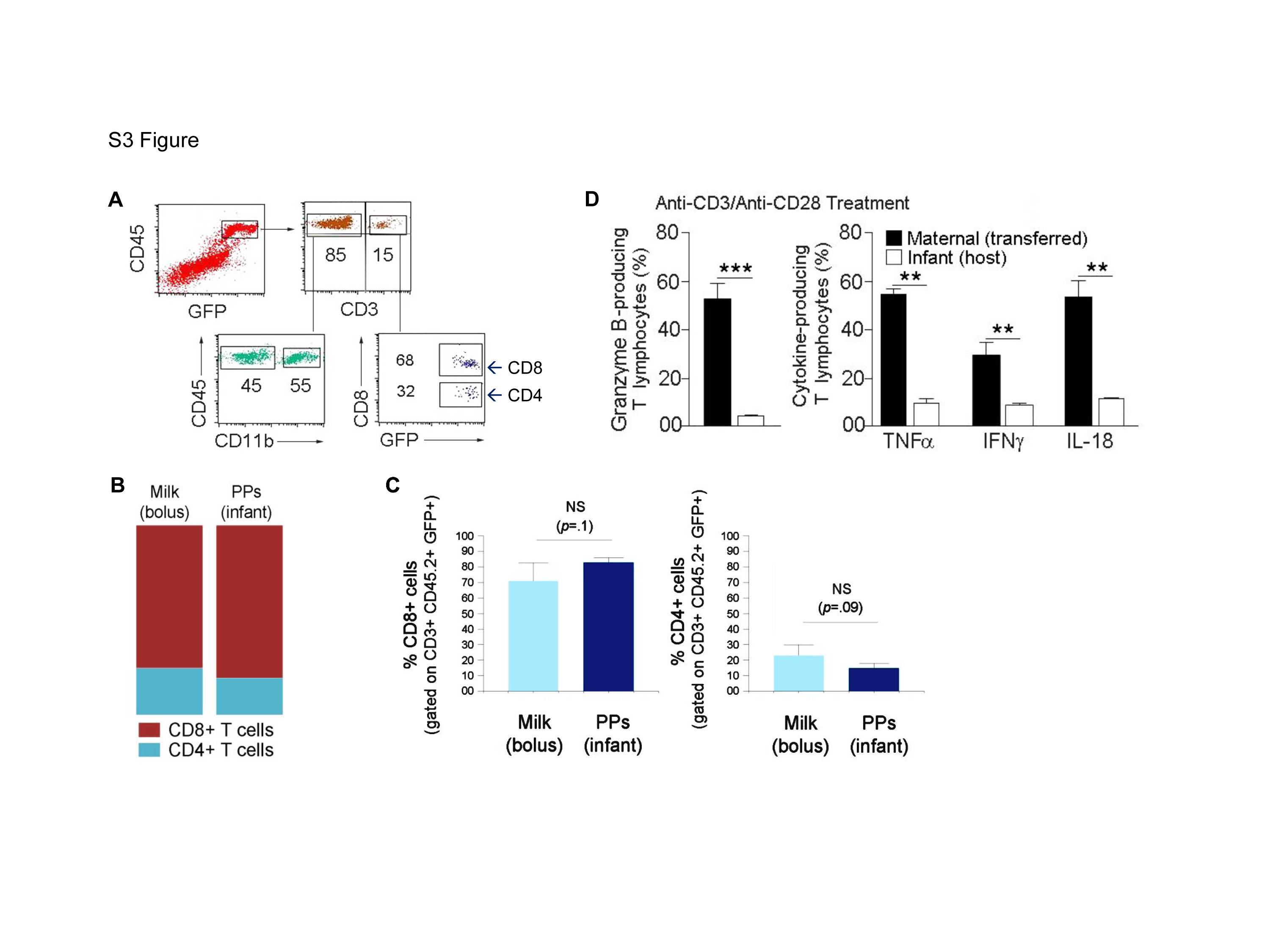

Supplement: S3 Fig — (A) Using similar orientating and specific gates as previously described by Faucher et al., [61], leukocyte types such as myeloid cells (CD11b) and T lymphocyte (CD3) subsets were identified in milk bolus. (B) Bar graphs show the average contribution of each T cell subset to CD3+GFP+T cells in milk bolus and PPs. (C) Bar graphs show the % of each T cell subset gated on total CD3+ GFP+ cells in milk bolus vs. PPs. Data are shown as means of % cell subset gated on CD3+GFP+ T cells SD. Error bars represent SD. Data were obtained from 4 experiments using a total of 16 (milk bolus) and 25 (PPs) animals with a combined material of 3–5 (milk) and 5–8 (PPs) mice per experiment. (D) PPs from CD45.1 pups foster-nursed by CD45.2 congenic dams for 18 days were collected, put into cell suspensions, and processed for T cell purification. Enriched T lymphocytes were activated with anti-CD3/anti-CD28 Abs for four days in the presence of 200U/ml of IL-2. T cell responses were examined by intracellular staining and analyzed by FACS. Bar graphs show % granzyme B- and cytokine-producing maternal (transferred by breast milk feeding, black) vs. infant (host, white) T lymphocytes. Data obtained from three experiments are shown as means of CD3+ T cells ± s.e.m. Error bars represent s.e.m. P < 0.05 was considered significant, **P < 0.005, ***P < 0.0005using student’s two-tailed t test. (TIF) [file pone.0156762.s003.tif]
